# A Comprehensive Survey on RF Energy Harvesting: Applications and Performance Determinants

**DOI:** 10.3390/s22082990

**Published:** 2022-04-13

**Authors:** Hafiz Husnain Raza Sherazi, Dimitrios Zorbas, Brendan O’Flynn

**Affiliations:** 1 School of Computing and Engineering, University of West London, London W5 5RF, UK; sherazi@uwl.ac.uk; 2 Tyndall National Institute, University College Cork, T12R5CP Cork, Ireland; brendan.oflynn@tyndall.ie; 3 Department of Computer Science, School of Engineering & Digital Sciences, Nazarbayev University, Nur-Sultan 010000, Kazakhstan

**Keywords:** energy harvesting, RF powered wireless networks, RF-harvesting techniques, energy propagation models, RF circuit design, MAC protocols for RF power harvesting

## Abstract

There has been an explosion in research focused on Internet of Things (IoT) devices in recent years, with a broad range of use cases in different domains ranging from industrial automation to business analytics. Being battery-powered, these small devices are expected to last for extended periods (i.e., in some instances up to tens of years) to ensure network longevity and data streams with the required temporal and spatial granularity. It becomes even more critical when IoT devices are installed within a harsh environment where battery replacement/charging is both costly and labour intensive. Recent developments in the energy harvesting paradigm have significantly contributed towards mitigating this critical energy issue by incorporating the renewable energy potentially available within any environment in which a sensor network is deployed. Radio Frequency (RF) energy harvesting is one of the promising approaches being investigated in the research community to address this challenge, conducted by harvesting energy from the incident radio waves from both ambient and dedicated radio sources. A limited number of studies are available covering the state of the art related to specific research topics in this space, but there is a gap in the consolidation of domain knowledge associated with the factors influencing the performance of RF power harvesting systems. Moreover, a number of topics and research challenges affecting the performance of RF harvesting systems are still unreported, which deserve special attention. To this end, this article starts by providing an overview of the different application domains of RF power harvesting outlining their performance requirements and summarizing the RF power harvesting techniques with their associated power densities. It then comprehensively surveys the available literature on the horizons that affect the performance of RF energy harvesting, taking into account the evaluation metrics, power propagation models, rectenna architectures, and MAC protocols for RF energy harvesting. Finally, it summarizes the available literature associated with RF powered networks and highlights the limitations, challenges, and future research directions by synthesizing the research efforts in the field of RF energy harvesting to progress research in this area.

## 1. Introduction

The IoT [1] is a research area that has been attracting the attention of research communities around the globe for the past decade. The IoT can generally be seen as a networked system of smart interconnecting objects (i.e., sensors, actuators, machines, smart phones, tablets, etc.). These devices have the ability to sense different types of data (i.e., temperature, pressure, light, acceleration, and so on) from the environment, process it in real-time, and transmit it to the cloud for analysis in order to react to some events of interest. As per some recent statistics forecast by industrial leaders, approximately 125 billion connecting devices are expected to be deployed by 2030 as a part of the IoT, which represents more than sixteen times the world’s population [2]. It is estimated that the IoT market will be worth in the region of USD 1.1 Trillion by the year 2023 and will enable industry activity in this area to rapidly scale up their business operations and associated Research and Development activities [2]. This massive explosion of smart devices will not only pave the way towards ubiquitous services in different application domains (i.e., Industry 4.0, smart home, smart agriculture, smart supply chain, and smart city, to name a few) [3], but will also open up research activities addressing associated research challenges (e.g., energy efficiency, long radio coverage, optimum throughout and latency, to name a few) in all these domains [4,5,6,7,8]. IoT devices are, in general, battery-powered in nature and the energy-intensive system operation being carried out in the aforementioned use cases requires that the batteries be recharged/replaced after a certain period, which is expensive in terms of both cost and labour [9]. Moreover, these batteries are also a source of carbon emission with adverse environmental effects [10]. Thanks to the potential of renewable energy sources [11,12] available within the specific deployment environment, the surplus harvested energy availability could circumvent power issues in smart devices to significantly prolong the network lifetime to make them ‘deploy and forget’. Table 1 summarizes different renewable energy sources along with their harvesting potential and conversion efficiencies [13].

**Figure 1:**
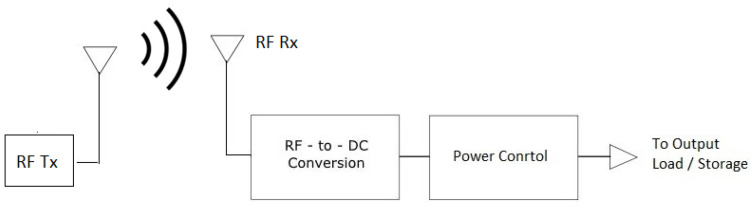
RF energy harvesting architecture.

RF energy harvesting, is one of a number of renewable energy sources which can provide an alternative to batteries as a power source for wireless sensors. RF energy refers to the amount of energy harvested from the RF signals (i.e., both ambient and dedicated) emitted by a transmitter and received by the harvesting circuit to generate an electrical energy as shown in Figure 1. RF energy harvesting offers a promising solution for energy constrained networks (such as the IoT) by converting RF signals into electrical energy which can be used to provision the energy needs of the smart devices in order to prolong their lifetime. In a typical RF energy harvesting use case, a radio signal with a frequency range between 3 kHz and 3 GHz could be employed as a medium to provide the electromagnetic energy from the transmitter source to the receiver [14].

RF energy harvesting as an energy transfer mechanism can provide a comparatively sustainable supply with easy availability, when compared to other energy harvesting methods (such as vibration, solar, or thermal), and can help meet the energy requirements of IoT-oriented energy-constrained networks. This can help meet the energy requirements associated with communication primitives, sensing, processing, and transmission operations. Hence, such RF energy harvesting has numerous applications in several domains and as a result several manufacturers are developing commercial solutions to address this market need. Some examples are the TX91501b Powercaster transmitter [15], the Powerspot [16], and the Cota system [17,18]. A number of experimental evaluations of RF energy harvesting systems with a variety of harvesting sources are summarized in Table 2, where it is clearly evident that the levels of energy generated significantly varies over the different distances and the strength of the source RF signals, which is typically in the order of μW which is generally sufficient to power up various low-power IoT devices.

**Methodology:** This article aims to address three main objectives. First, we provide an overview of the applications of RF power harvesting with their performance requirements and develop an understanding of the different harvesting techniques along with their power densities to familiarize the reader with the baseline performance requirements and achievable power densities by employing different RF harvesting techniques (Section 2 and Section 3). Second, we provide an analysis on the state of the art covering the most critical areas that affect the performance of RF energy harvesting systems and how the recent developments have contributed towards performance optimization focusing on the best design considerations, innovative techniques, and novel architectures (Section 4, Section 5, Section 6 and Section 7). Third, we highlight a number of limitations and research challenges to be addressed so as to obtain the optimum performance levels for IoT devices and to pave the future directions with research potential for the new entrants in this domain (Section 8).

The growing interest in RF energy harvesting, being a promising renewable energy solution, has also identified a number of technical challenges associated with the energy harvesting process (see Section 8.1), including antenna design for transmitters and receivers, rectifiers, and matching networks. There are a limited number of studies presently available, aimed at reviewing the current state of the art in the literature on RF energy harvesting (as shown in Table 3), but these studies do not give a clear view of the performance determinants for RF power harvesting systems. In general, the reviews are focused on specific aspects of the RF energy harvesting domain (such as hardware design issues [19,20], RF powered wireless networks [21], circuit and protocol design [22], harvesting sources [23], trade-offs and design methodologies [24,25], and conceptualization [26]). Moreover, many of the surveys available in the area are quite old and the most recent contributions are consequently unreported for the researchers currently working in this popular area. To the best of the authors’ knowledge, no comprehensive study is currently available highlighting the critical areas that directly influence the performance of RF energy harvesting systems.

**Contributions:** The present study achieves the following four-fold contributions:

- We overview a range of application domains benefiting from RF energy harvesting to achieve an optimum performance level, where we outline a number of performance requirements with respect to each of the application domains, in order to familiarize the readers with the baseline performance requirements that are expected to be met by RF energy harvesting systems.
- We investigate different RF power harvesting techniques along with their average and peak power densities in order to assess their suitability and limitations for a number of use cases in different application domains.
- We thoroughly study the performance determinants for RF power harvesting systems that critically influence the performance of these systems. This includes the evaluation metrics for RF energy harvesting and energy propagation models that affect the performance of RF-based systems. In addition, there is an analysis of rectenna architectures and the power conversion efficiency associated with different rectenna design approaches, and medium access protocols to support energy exhaustive operation in RF powered networks.
- We highlight the open issues, challenges, and lessons learned from recent implementations, and future research directions by synthesizing the research efforts that have been put in place in the field of RF energy harvesting in recent years.

This study aims at providing a clear road map for researchers to help them identify the relevant design considerations and research challenges that are crucial to the performance of RF energy harvesting systems.

**Organization:** The remainder of this article is organized as follows: Section 2 overviews the prospective applications of RF power harvesting along with their performance requirements. The RF harvesting techniques widely adopted by the industry along with their average and peak power densities are investigated in Section 3. The evaluation metrics for RF energy harvesting systems that effectively evaluate the performance of these systems are summarized in Section 4. Similarly, Section 5 presents the energy propagation models that affect the performance of RF based systems. Section 6 surveys different rectenna architectures, covering an in-depth analysis on the power conversion efficiency with different rectenna design approaches being one of the key performance criteria. Moreover, Section 7 presents an overview of MAC protocols specifically designed for RF energy harvesting systems. Section 8 highlights the open issues, challenges, and lessons learned from recent implementations, and future research directions by synthesizing the research efforts in the field of RF energy harvesting. Finally, concluding remarks are provided in Section 9.

## 2. Applications of RF Energy Harvesting

RF energy harvesting has plays a role in a number of application domains to achieve energy-efficient operation for a range of use cases. According to a report [42], energy sustainability and integrated services for automation are some of the main driving factors for a majority of applications in different domains. This section aims at reviewing the state of the art specifically targeting the most prominent applications where RF energy harvesting systems have been designed to meet a range of performance requirements (as outlined in Table 4) to achieve a unique set of objectives for the following applications.

### 2.1. Ubiquitous Internet of Things

The Internet of Things [1] is paving the way forward towards ubiquitous services in different application domains. This technological advancement simultaneously gives birth to a new set of challenges towards achieving energy optimal IoT operation [43]. Choudhary et al. [44] emphasize the need for power harvesting in a range of IoT applications and outline a number of energy harvesting technologies, methodologies, and architectures. Their work further surveys different energy harvesting sources and techniques for the researchers working in this domain. Ref. [45] is another attempt towards presenting a clear vision of green IoT, where IoT nodes are designed for extremely low energy consumption and are fed through renewable energy solutions. The authors aim to describe the current state of the art and provide insights into energy-saving practices and strategies for green IoT. The major contribution of this publication is the review and discussion of the substantial issues enabling hardware green IoT to focus on green wireless sensor networks and green radio-frequency identification. In Ref. [46], the authors advocate that ambient energy harvesting from the environment could be the only viable option for prolonging the lifetime of large scale interconnected networks.

### 2.2. Industrial Automation

Being a key enabler in the evolution of Industry 4.0, energy is a vital component in the integration of computational processes with the physical processes in a smart production environment. In such an environment, a variety of smart devices are deployed for the monitoring of physical industrial processes and these are continuously engaged in energy exhaustive operations. For example, the frequent sensing and transmission of important data of interest to the expert system is critical to Cyber-Physical Systems (CPS) [47]. However, this is known to be a power-hungry operation for these devices which are primarily battery-powered in nature [48]. Hence, the critical aspect of these low power devices is the energy efficient operation and optimization of energy management and harvesting mechanisms.

Tahir et al. [49] present a low-cost scheme for measuring the RF signals on the production floor with the aim of optimizing the manufacturing operation within a smart industry. The RF energy can then be harvested at several locations of the manufacturing line and the harvested energy supplied to Micro-Electro-Mechanical Systems (MEMS) and sensors installed on the production line. As such the RF harvesting system can reduce the maintenance labor and operational costs associated with frequent battery replenishment. Similarly, Tang et al. [50] outline a range of energy harvesting technologies suitable for industrial automation and investigate the energy consumption of small sensor devices deployed across the production floor. The authors evaluate the energy harvesting potential that can be harnessed from the industrial processes. Finally, a number of energy harvesting technologies and prototypes are reviewed in different areas with a special emphasis on their use for production machinery in industrial applications.

Similarly, a collection of tiny nodes capable of sensing the environment, performing simple computations and supporting wireless communications to accomplish a monitoring task can be referred to as Wireless Sensor Networks (WSNs). Since their emergence a number of decades ago, WSNs have been adopted in a variety of use cases including, but not limited, to Smart Homes [51], Smart Healthcare Systems [52], Intelligent Transportation Systems [53], Disaster Management Systems [54], and Continuous Video Surveillance Systems [55]. The energy consumption of such wireless sensor nodes varies depending on the use case requirements. To ensure a sustainable system design, RF power harvesting capabilities can be incorporated in to the WSN system design. To this end, Ref. [56] presents a practical scheme for RF energy harvesting and management for WSNs based on an Improved Energy Efficient Ant Based Routing Algorithm (IEEABR) and taking into account the energy consumption of the sensor nodes and availability of RF energy. The authors also cover the measurement statistics of RF power density, estimation of received power, storage of harvested power to a storage media, and associated management of RF power harvesting in the context of WSNs.

A similar work [57] outlines the requirements and fundamental principles for powering up WSNs by RF energy harvesting. The harvesting feasibility and the power density is evaluated to conclude that RF energy harvesting is most appropriate to feed small-sized sensors. Similarly, Ref. [58] overviews the magnitudes of various ambient energy harvesting sources for WSNs. They argue that 10–100 μW, although small, is an ample order of magnitude for a range of applications in WSNs. Meanwhile, Ref. [59] is another work to implement RF energy harvester in Mica2 nodes with an adopted duty cycle to harvest sufficient amount of energy to feed this sensor. Sample et al. [60] demonstrate that the power transmitted from a 4 km distant TV station is capable of feeding a temperature sensor. Moreover, a 30 cm by 20 cm antenna is sufficient to harvest around 60 μW yielding a power density of about 0.1 μW/cm${}^{2}$.

### 2.3. Healthcare Informatics

With the digital transformation of patients data from manual spreadsheets to complete web-based healthcare solutions, healthcare informatics have rapidly been evolved over the last few decades. Web-based healthcare solutions combined together with IoT driven smart systems enable universal accessibility of data for doctors, nurses, paramedics, healthcare staff, and laboratories across the globe [61]. In this application domain, there have been numerous implementations of RF energy harvesting sensor nodes for Wireless Body Area Networks (WBAN) [62,63,64,65] and medical wearables [66,67,68], including systems for patient health monitoring and wireless smart injection mechanisms [69]. Thanks to the integration of RF energy harvesting capability, low-power medical devices can deliver real-time data sets and on-demand services are feasible due to the associated battery-free design [26]. For example, Ref. [62] et al. developed an implementation of a WBAN sensor comprising of a small triple band antenna, a DC energy management and storage module, a sensing and communication module, and a micro controller to enable autonomous behavior. To convert RF energy harvesting into DC power, a triple band rectifier was designed for low power applications which successfully achieves 59% conversion efficiency for an input power of −10 dBm. The proposed sensor is compact and suitable for self monitoring of human body in healthcare informatics. Similarly, Ref. [66] provides a comprehensive review on both the scientific literature and commercially available RF power harvesting devices in wearable healthcare.

### 2.4. Radio Frequency Identification (RFID)

Radio Frequency Identification (RFID) [70] is one of the mature short-range wireless technologies in which the data is digitally encoded in small radio transponders (also known as tags or smart labels). RFID belongs to the technological family of Automatic Identification and Data capture, where tags need not be in line-of-sight for reading the data within the supported range. As RFID is based on a near-field technology, it is an efficient RF power and data transmission system. Numerous works have been identified using RF power harvesting in a variety of RFID applications [71,72,73,74,75,76,77,78]. Mhatre et al. [71] describe a three-phase energy harvesting system where they first describe a dual band antenna capable of supporting a large frequency response ranging from 900 MHz to 2.45 GHz and fair radiation patterns. In addition, the authors describe a rectenna for converting the received RF energy to DC voltage. The integration system provides a conversion efficiency of 30% at 2.4 GHz.

Moreover, Ref. [72] overviews the RFID operation in more general terms and describes in detail a range of different RFID tags. The need and dynamics of RF power harvesting for active RFID tags are identified and highlighted. In Ref. [73], RF energy harvesting is identified as a source of green energy, suitable for a range of sensor applications in harsh environments. Another study [74] provides a comprehensive review on the advancements on the implementations of RFID sensors with a particular emphasis on RF power harvesting, describing recent progress in the state of the art, and commercially available solutions with innovative applications. Olgun et al. [75] discuss RFID rectenna design with the aim to harvest energy for powering up RFID from ambient energy sources operating in the 2.45 GHz ISM (Industrial, Scientific, and Medical) band. Finally, Bukhtiar et al. [77] describe an RF energy harvesting system based on automatic input tuning for far-field RFID tags. A high quality factor LC-matching network is employed to boost the voltage of received RF power and then a proof of concept system is simulated in 0.13 μm CMOS demonstrating a conversion efficiency of 50%.

### 2.5. Smart Buildings and Structural Health Monitoring

A smart building generally refers to a structure employing automated processes to control the building operations (such as heating, ventilation, lighting, air conditioning, and security, to name a few) in order to better understand the linkage between a building and its occupants to improve the living experience of inhabitants and energy efficiency of the building [79]. Structural health monitoring aims to foresee the potential risks associated with the building structure before they actually cause any damage. Several scientific studies [80,81,82,83,84] discuss the usefulness of RF energy harvesting to provide energy to sensors deployed in the building structures because of their high battery maintenance costs, as the sensors are often installed in inaccessible places within a building or structure being monitored (such as bridges and tunnels) [82]. Ref. [80] describes the design of a rectenna for RF energy harvesting employing a compact wide band antenna operated at targeted 8–18 GHz frequency bands yielding a DC output of 1.8V that can convert to 5–8 mV at 20 dBm to power sensors in structural health monitoring applications. However, the gains achieved through such wide band antenna is relatively lower because of the power distributed across the wide frequency band.

Similarly, Ref. [81] demonstrate an implementation of sensors as a part of CPS to realize structural health monitoring for smart buildings. The networks comprises of battery-free LoRaWAN prototypes. One of them is powered by RF energy harvesting system operated in the unlicensed ISM 868 MHz band. Extending the work in [81,82] exploits battery-free LoRa motes directly fed by RF power harvesting to sense and report temperature and relative humidity levels in structural health monitoring of smart buildings over a distance ranging from several meters to kilometers. In Ref. [83], the authors address the design and characterize a 66 cm${}^{2}$ sized rectenna operating at 868 MHz to power structural health monitoring sensing and communications applications. Lastly, Ref. [84] surveys recent advancements in the ambient energy sources and power harvesting methodologies used to feed sensors in structural health monitoring applications with a special emphasis on RF power harvesting applications.

## 3. Dedicated vs. Ambient RF Energy Harvesting

The choice of wireless power harvesting technique to use in a particular application should be considered carefully as it directly impacts the performance of RF power harvesting systems and depends greatly on the requirements of the applications. Harvesting energy from RF power sources differ from other harvesting techniques (such as solar, wind, thermal, and vibrational) primarily because of the following characteristics. RF sources are capable of providing a constant and controllable level of power over several distances particularly in fixed-size RF energy harvesting networks where the distance between transmitter and receiver is static and a known parameter. As the exact amount of harvested power depends on the distance of the receiver from the source, network nodes installed in different locations may observe significant difference in the amount of harvested energy compared with other nodes in the same network [31].

RF power harvesting can be classified into two different types: ambient and dedicated RF power harvesting. RF energy harvesting associated with the natural sources present within the environment can be referred to as ambient energy harvesting, and can generate energy from sources such as digital TV, WiFi, and GSM signals. Dedicated RF sources can also be used to harvest a required amount of energy for an application where ambient RF sources do not provide sufficient energy to meet the application requirements. This section describes the methodologies and prototypes from literature employing both ambient and dedicated RF energy harvesting techniques with the objective being to highlight the pros and cons associated with these techniques in different environments.

### 3.1. Dedicated RF Energy Harvesting Sources

Dedicated RF energy harvesting techniques involve installing dedicated sources of energy to enable power harvesting within the environment. These dedicated sources can employ license-free ISM bands for power transfer. The commercially available powercaster transmitter [15] operating at sub-GHz band with 1–3 W transmission power is a good example of a dedicated RF source. However, this kind of dedicated RF harvesting system can incur high costs, especially in the case of large scale networks where large quantities of such sources may need to be installed. This might make the technology inappropriate for some application deployments. Moreover, the maximum output power transmitted by these RF sources is governed by local frequency regulation agencies such as Federal Communications Commission (FCC) [85] or general ISM regulations to avoid interference with other critical applications and ensure public safety.

There are a number of prototypes and implementations of dedicated RF harvesting sources available in the literature. For example, Zorbas et al. [86] modeled a network with sensor nodes capable of harvesting energy from RF sources and associated dedicated energy transmitters to power them up to enable sensing and communication of data. The authors identify the factors affecting the energy consumption of these nodes (such as network density and the number of transmitted packets). Moreover, they also developed a method for multi-hop energy transfer between the nodes. An interesting contribution coming from this work is the cost analysis presented therein to establish if the cost incurred to design these RF energy harvesting nodes can be compensated with a corresponding reduction in the level of maintenance costs for the system due to the presence of energy harvesting capabilities.

Similarly, Radhika et al. [87] developed an RF powered sensor platform where the energy could be harvested from either dedicated power sources or ambient RF powered sources. The authors describe a set of requirements with a dedicated chip designed for the sole purpose of power harvesting and conditioning. The designed hybrid system is powered employing sub-GHz band frequencies for dedicated radiations demonstrating a system efficiency of 9.1% with 90 s transmission interval for dedicated power source and a maximum efficiency of 44% with 9.75 k resisting load in ambient harvesting source. Another interesting work [88] considers wireless powered networks with dedicated power beacons and multi-user nodes scenario where multiple user nodes are harvesting energy from power beacons and compete for the transmission. The user nodes have no other power source hence they are solely based on the energy harvested from the power beacons and are assumed to be in harvesting mode, consumption mode, or idle mode. The authors propose a charge scheduling scheme taking into account the behavior of power beacons and user nodes to maximize the total harvested power.

Following the similar lines, Lakshmi et al. [89] come up with an efficient RF power harvesting scheme where multiple dedicated RF harvesting sources were deployed to fulfill the energy demands of a network. The work first focuses on the optimal location and the number of these dedicated transmitters. The authors then devise a utility function describing the placement of energy transmitters prioritizing the energy requirement of relay nodes and maintaining the minimal level of energy to provide for all the sensor nodes within a network.

Furthermore, Li et al. [90] propose wirelessly powered WSNs where the sensor nodes are harvesting sufficient power from a dedicated RF power source to share the information with the nearby communications data sink. The authors propose two different operation modes: Frequency Division Multiplexing (FDM) to harvest energy and send information simultaneously over an orthogonal frequency band, and Time Division Multiplexing (TDM) to enable both energy harvesting and information transmission to be incorporated in the same frequency band but in different time slots. Finally, various RF power transmission schemes were introduced [91,92,93] considering dedicated mobile transmitters with an intention to feed different WSN applications.

### 3.2. Ambient RF Energy Harvesting Sources

Ambient RF harvesting refers to the technique of harvesting power from ambient sources which are freely available electromagnetic radiations in the environment in which the sensor node is deployed. There are typically many different radio signals in the air which are targeting different primary objectives (such as WiFi for the Internet connectivity, Terrestrial Television and Satellite for the broadcasting of TV channels, and Global System for Mobiles (GSM) to enable mobile phone connectivity for voice, messaging, and data) that can be employed to harvest a small amount of energy. It is important to note that the level of magnitude and the predictability of harvested energy in the case of ambient energy harvesting is comparatively lower than that which is available from dedicated RF harvesting sources, but is still sufficient to provision sensor nodes in a variety of applications [94]. The transmitting power of all these ambient sources varies significantly, from $10^{6}$ W transmitted from TV towers to 0.1 W from WiFi access points and mobile communications. Table 5 presents the average and maximum power densities of ambient RF power sources available at different frequencies taking into account the summation of all the spectral peaks in an entire band. The research community has extensively worked dealing with different aspects related to ambient RF harvesting sources.

For example, Pinuela et al. [95] evaluated the harvesting potential at different ambient RF power levels and frequencies on the streets outside all metro stations across London City. Consequently, four harvesters were designed covering four different frequencies from the largest RF sources available in the metropolitan area such as DTV, GSM900, GSM1800, and 3G within the ultra high frequency spanning 0.3GHz to 3GHz. Prototypes were designed for each individual frequency band. The results demonstrate that almost half of the underground stations were deemed suitable for RF power harvesting when employing their designed prototypes. The output dc power density comparison was performed across all harvesters to demonstrate that RF harvesting can be competitive with respect to other harvesting methodologies.

Following similar lines, Ref. [96] studied the feasibility of various ambient harvesting energy sources through a preliminary assessment of power density and received power in a suburban area. The results demonstrated that received power available (i.e., −25 dBm from 800 MHz) from a mobile base station is 13 dB higher than that available from digital TV broadcasting. The authors designed and tested a circuit for RF–DC conversion and claimed that a capacitor of 0.1 F could be charged up to 320 mV in 65 h from this ambient RF source present in a suburb. Another work [97] presents the measurement of power density of ambient RF harvesting sources and argue that density in broadband (1–3.5 GHz) is in the order of 63 μW/m${}^{2}$. Two different systems have been considered to harness the ambient RF energy; a broadband system without a matching circuit and a narrow-band system with a matching circuit. The results conclude that the harvested energy is not sufficient enough to directly feed a device but could be stored in a super capacitor or a micro battery to be utilized further.

Moreover, Ref. [98] reviews various ambient energy harvesting mechanisms with a special emphasis on RF power harvesting where they investigate a far-field low power density technology enabled prototype of embedded micro controllers powered by ultra high frequency digital signals (512–566 MHz) where a broadcasting tower is 6.3 km away from the harvester. A dual-band ambient RF energy harvester is employed at 915 MHz and 2.4 GHz and an on-body harvester is also tested at 450 MHz to verify the potential of such harvesters for smart skin applications. Furthermore, Ref. [99] estimates the ambient RF and microwave power that can be harvested through the omni-directional electromagnetic sources studying the underlying microwave rectification at low power (i.e., −30 dBm or even lower) levels. A theoretical model is derived to accurately predict the rectifier’s efficiency and the insertion losses of matching networks. The results provide a comparison of different microwave rectifiers with the proposed model which clearly exhibits a 10% improvement in the efficiency of the proposed model as compared to existing solutions.

Finally, Ungan et al. [100] have developed the concept of rectifying the energy from low power ambient sources to feed microsystems. The authors designed a circuit and gauged the efficiency of the RF harvesting system. An ambient radiation source of 1 μW and an impedance of 50 Ohm on 300 MHz was employed to match the impedance and resonant circuit transformation with a high quality factor of 25.

Ambient harvesting can further be classified into static and dynamic ambient harvesting. The following subsections discuss both types of RF ambient harvesting with an example for each type of implementations.

Static ambient RF power harvesting refers to the energy harvesting from RF sources transmitting relatively stable power over any period of time (such as television and radio towers). Although the energy harvested by static ambient RF sources is more predictable compared to the energy harvested through dynamic sources, it can still undergo short or long term fluctuations due to varying service schedules and signal fading. As the power density of static ambient sources is small at different frequency bands, a high gain antenna is required for all these frequency bands and the rectifier should also be designed keeping in view the wide band spectrum. For example, Ref. [101] presents the performance analysis of a sensor employing stochastic geometry when powering it with static ambient RF energy harvesting sources and demonstrates that higher RF harvesting rates could be attained by the sensors when the distribution of RF sources shows stronger repulsion.

Dynamic ambient RF sources are those working periodically and which transmit varying powers at different times. This includes radio signals such as WiFi and primary users in cognitive radio networks. Hence, power harvesting systems generating energy from dynamic sources should be adaptive in nature, employing some kind of intelligence to establish as to where the optimum frequency for power generation is in different frequency bands. Here, Ref. [102] provides a good example of RF harvesting from dynamic ambient sources in a cognitive radio network where secondary users are able to harvest RF energy from the primary users nearby. The wireless nodes can then utilize this energy to transmit data when the primary users nearby are in idle mode or are sufficiently far away from the primary users. To summarize, there are certain pros and cons associated with both dedicated and ambient RF sources which decides their applicability in a range of use cases. However, small power densities associated with ambient RF sources can limit its suitability for some applications in different domains. Similarly, achieving longer distances between RF transmitters and harvesters is still a challenge as the range of energy transfer directly influences the performance of the harvesting mechanism. Moreover, dedicated sources are better in terms of achieving higher conversion efficiency but the higher CAPEX (i.e., Capital Expenditures) cost associated with the installation of dedicated RF power chargers may be a concern. Furthermore, site selection associated with the installation of dedicated chargers can play a significant role in optimizing the range between transmitters and receivers in a network architecture definition. Finally, antenna and rectifier design for static ambient RF harvesting systems should be considered in order to maximize a stable amount of RF energy being harvested.

## 4. RF Energy Harvesting Evaluation Metrics

In this section, the key performance metrics associated with the evaluation of RF power are described in the context of energy harvesting. There are a number of parameters that should be evaluated which can play a decisive role in the performance of an RF energy harvesting design and implementation. These evaluation metrics are defined by the underlying application requirements and can change according to the application scenario. The most relevant metrics used for evaluating RF power harvesting systems include standard parameters such as the range between transmitter and receiver, the efficiency of the harvesting system, the harvester sensitivity, the resonance factor of a resonator, and the output power obtained at the receiver [104]. However, evaluating the trade-offs between the metrics (e.g., the operation range and efficiency) is vital in the comparison of these metrics. Moreover, some other related factors such as bulk manufacturing availability, maturity of fabrication process, and low cost design can also be factors to consider in the selection of an RF energy harvesting technology.

Following are some of the key evaluation metrics in the domain of RF energy harvesting which can significantly impact on the efficiency of the harvesting system. The main objectives are to achieve the optimal power conversion efficiency, output power, and receiver sensitivity of the RF harvesting systems and the choice of optimal operational frequency of the incident signal is critical in RF energy harvesting systems.

### 4.1. Range and Frequency

**Figure 2:**
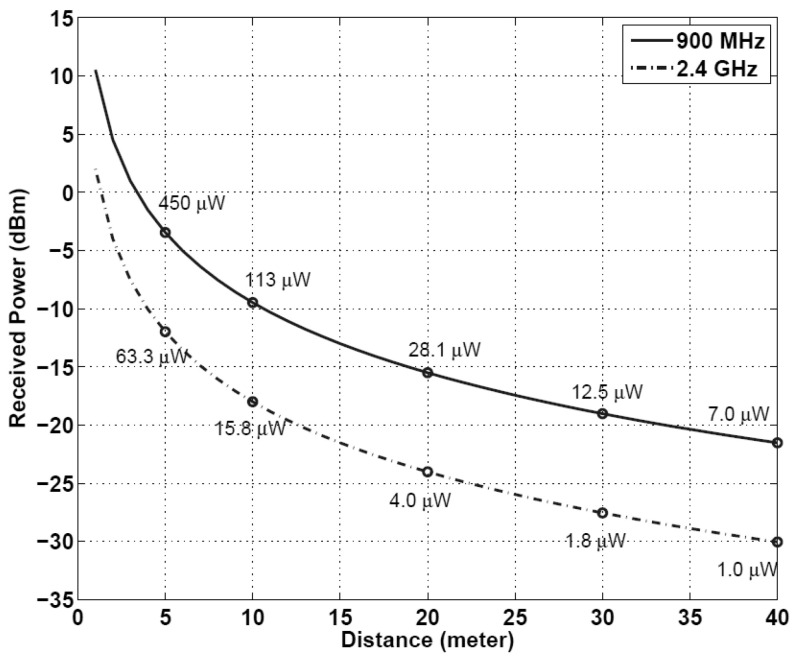
The impact of distance on the received power in RF power harvesting.

The operating distance or range between the energy transmitter and receiver is one of the key RF power performance metrics to be considered (see Figure 2). This is related to the operational frequency of the system in question. For example, systems which are transmitting at higher frequencies suffer more from attenuation as compared to low frequency transmissions due to the longer wavelength when passing through the wireless medium. However, low frequency signals penetrate deeper through matter compared to their counterparts. For instance, the transmitting frequencies should not exceed a few megahertz in case the target application of RF power harvesting is an implantable device.

### 4.2. Conversion Efficiency

**Figure 3:**
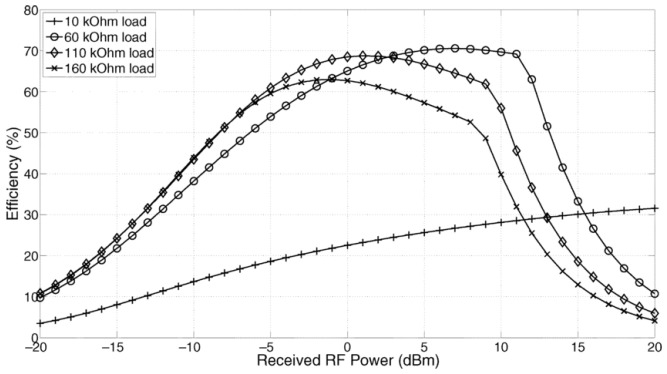
Power conversion efficiency vs. received RF power against different loads.

Power conversion efficiency (PCE) is another critical metric when comparing performance of RF harvesting systems. This is the ratio of the power applied to the load versus the power gained by the antenna. Here, the RF-DC power conversion efficiency not only refers to the efficiency of a rectifier, but the voltage multiplier and the storage element as well. RF transmission losses are not usually considered in this conversion efficiency calculation. This can simply be expressed as the ratio of the power delivered to the source to that of retrieved power as follows [104]:

(1) $$\eta_{PCE}=\frac{P_{load}}{P_{retrieved}}.$$

where $P_{load}$ is the power applied on the load and $P_{retrieved}$ is the harvested power at the antenna. A comparison of power conversion efficiency against different levels of received RF power can be seen in Figure 3 when different levels of loads are applied (such as 10 kΩ, 60 kΩ, 110 kΩ, and 160 kΩ) using a Schottky diode with a conventional CMOS rectifier topology.

### 4.3. Resonance Factor

Resonance occurs when a system is able to store and easily transfer energy between different storage modes, such as Kinetic energy or Potential energy (as in case of a simple pendulum). Most energy harvesters are designed to work at resonance frequency in order to obtain maximum output power. Resonant frequency is the oscillation of a system at its natural or unforced resonance. The larger the antenna size, the lower the resonance frequency. Hence, resonance factor (also known as *Q* factor) is defined as the generic dimensionless value describing the strength and bandwidth of resonance. The *Q* factor represents the increase in the peak voltage when the system needs to resonate on resonant frequency. This derives the expression of *Q* factor as follows [105]:

(2) $$Q=2\pi \frac{E_{stored}}{E_{dissipated}}$$

where $E_{stored}$ is the total stored energy and $E_{dissipated}$ is the dissipated amount of energy per cycle. Hence, it can be inferred that a high *Q* factor refers to the narrow resonant bandwidth but high voltage gain. Moreover, Equation (2) also implies that *Q* factor is inversely proportional to the amount of dissipated energy during each discharge cycle.

### 4.4. Sensitivity

Sensitivity is defined as the minimum amount of incident power required for triggering a system’s operation. It is the ability to harvest energy and operate at a low power density. The greater the sensitivity of a harvesting system, the better the conversion efficiency due to the strength of incident signal and hence the performance of the system. Sensitivity can be quantified by the following expression [104]:

(3) $$S\left(dBm\right)=10\log_{10}\frac{P_{min}}{1\;\mathrm{mW}}$$

where $P_{min}$ refers to the minimum power a system is required to accomplish a task. It is pertinent to note that threshold voltage of CMOS influences sensitivity as lower threshold CMOS offers more sensitivity but on the cost of higher current leakages, ultimately compromising on the system efficiency.

### 4.5. Output Power

Output power is another key metric for the performance evaluation of power harvesting systems which is usually described in the form of DC power, a product of voltage applied by the load (*V*) and the current (*I*). The measurement of the load voltage demonstrates the performance of a system which depends on the load impedance. For example, in the case where the load is a sensor, the voltage (*V*) is more important than the current (*I*). Contrarily, in the applications incorporating LEDs or electrolysis, *I* is more dominant compared to *V*.

## 5. Energy Propagation Models

It is essential to evaluate the propagation characteristics of a radio system in order to precisely estimate the set of signal parameters that are needed to measure the quality and performance of an RF signal associated with a harvesting system. The analysis of signal propagation enables us to make an appropriate estimation of the signal characteristics. The accurate prediction of the behavior of a radio signal is critical to the system design for future RF harvesting systems. Moreover, propagation models are a low-cost alternative to physical site examination and measurement which makes optimum deployment more feasible by analyzing the propagation models for the deployment site [106]. This section reviews propagation models for different RF energy harvesting systems available in the literature.

### 5.1. Deterministic Models

It is pertinent to note that the amount of harvestable RF energy depends on the transmitting power of the source, the distance between the source and the harvester, and the wavelength of the RF signal transmitted and received by a receiving antenna. Deterministic models characterize the electromagnetic radiation propagation based on a set of deterministic parameters which are known in advance. The following are the two deterministic propagation models most widely considered for studying the propagation behavior of RF signals.

#### 5.1.1. Free-Space Model

The free space propagation model is a model which can help in understanding the behavior of RF signals. The transmitted power of an RF signal in free space can be evaluated based on the famous Friis Equation [107]:

(4) $$P_{R}=P_{T}\frac{G_{T}G_{R}\lambda^{2}}{{\left(4\pi d\right)}^{2}L}$$

where $P_{R}$, $P_{T}$, *L*, $G_{T}$, $G_{R}$, λ, and *d* are the received power at the harvester, the transmit power from the source, the path loss factor, the gain of the transmitting antenna, the gain of the receiving antenna, the wavelength of the RF signal, and the distance between the transmit antenna and the receiver antenna at harvester, respectively. The free space model is simplistic model assuming that there is only a single path between the transmitting and receiving antennas while this is not always the case.

#### 5.1.2. Two-Ray Ground Model

The two-ray ground model [108] is another propagation model that comes into play in the presence of scattering and reflection of the antecedent RF signal. Due to this scattering, the received RF signal reaches the harvester after passing through multiple paths and as such, the free space model is unable to provide accurate estimations. The two-ray propagation model takes into account both the line-of-sight signal and reflected signal to resolve this problem. Hence, the harvested RF power from transmitter presented by the two-ray ground model could be expressed as:

(5) $$P_{R}=P_{T}\frac{G_{T}G_{R}h_{t}^{2}h_{r}^{2}}{d^{4}L}.$$

while $h_{t}$ and $h_{r}$ are the heights of the transmitting and receiving antennas, respectively.

### 5.2. Probabilistic Models

Unlike deterministic models, probabilistic models deduce parameters based on distributions which allow more realistic modeling compared to deterministic parameters modeling. The Rayleigh model [109] is one of the more widely used and applicable probabilistic propagation models capable of representing the scenarios where a line-of-sight channel between the transmitter and receiver is absent. Hence, the transmit power of RF signals expressed by Rayleigh model could be as follows:

(6) $$P_{R}=P_{R}^{det}\times 10^{L}\times {\left|r\right|}^{2},$$

where $P_{R}^{det}$ indicates the received RF power estimated by the deterministic models in Equations (4) and (5). The path-loss factor *L* is defined as follows [109]:

(7) $$L\left(d\right)=\bar{L(d_{0})}+10alog\left(\frac{d}{d_{0}}\right)+G,$$

where *d* is the distance between the transmitter and the receiver, $L(d_{0})$ is the mean path-loss for a reference distance $d_{0}$, *a* is the path-loss exponent, and *G* is a zero-mean Gaussian distributed random variable with variance σ [109]. The cumulative amount of RF harvested power could be evaluated based on both the type of the network model and chosen RF propagation model. For instance, Ref. [110] considers the transmitters to be distributed spatially as per the Poisson process and characterize the distribution of RF power received at the harvester. They provide the theoretical estimation for the distribution of RF power received at the harvester taking into account the fading effects and path loss. The authors propose a probabilistic model based on that RF power distribution to find the probability of a node to transmit a packet after having a certain recharge time. The results taken from their analysis were encouraging and quite close to the simulation results that justified the accuracy.

Moreover, Ref. [111] models the electromagnetic energy harvested by RF radiations through the gamma process for energy harvesting systems. The characterization of the harvesting energy is achieved from the Nakagami-m model as a continuous-time stochastic process defining the signal reception in a number of frequency channels. The transmission policy is defined considering different fading conditions and a set of performance measures is presented to analyze the performance of harvesting system. Furthermore, Ref. [112] characterizes the distribution of RF power received by the harvester acknowledging that transmitters are spatially distributed through Poisson process. The authors demonstrate that the RF received power can be approximated by the summation of multiple gamma distributions with different parameters for scale and shape taking into account the shadowing and Rayleigh fading. The authors eventually derive the probability of a node to transmit a packet after a specific charging time. The numerical results demonstrated herein are quite close to the simulated ones which argues about the validity of the proposed system.

## 6. Rectenna Architecture for RF Energy Harvesters

**Figure 4:**
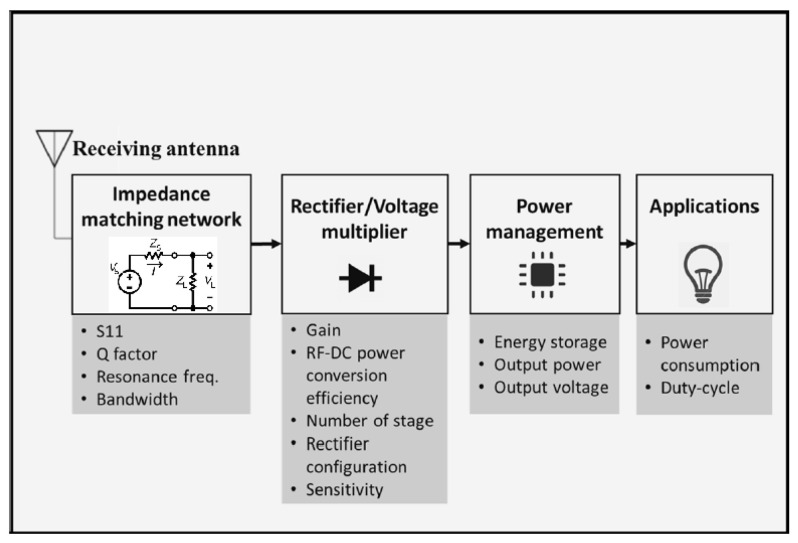
Rectenna architecture for RF power harvesting system.

The rectifying antenna (i.e., rectenna) circuit architecture is of central importance in the context of performance optimization of RF energy harvesting (see Figure 4). It serves to receive RF signals in a single frequency or a range of different frequencies and converts them into DC power. The quality of a rectenna can be gauged by measuring the conversion efficiency it offers while operating in different frequency bands [113]. This section covers different aspects of the rectenna design starting from considerations of an antenna design and then reviewing the state of the art available on both single and multi-antenna RF harvesters, impedance matching networks, and rectifiers. Moreover, the reader can refer to a related recent review on antenna technologies for ambient RF energy harvesting [114].

### 6.1. Antenna Design Considerations

The antenna is one of the integral components of RF power harvester which is primarily responsible for collecting the RF power from a transmitter to transfer it to the rest of the rectenna circuit (such as matching network, rectifier, and low pass filters) for conversion into DC power and feeding the load. Hence, the antenna design is of paramount importance in obtaining the maximum power transfer efficiency. There are certain challenges in the antenna design that can broadly be classified into conversion efficiency, form factor, and bandwidth. Some of the key design considerations for antennas are discussed throughout this subsection that could help to maximize efficiencies and achieve the desired performance objectives.

#### 6.1.1. Antenna Characteristics

Thanks to recent advancements in very large scale integration technologies, the electronic circuits and systems are consistently being miniaturized following the demands of consumer of compact size through the use of embeddable components including antennas. However, achieving this milestone is not straight forward and involves a trade-off as regards a range of parameters such as bandwidth, beamwidth, efficiency, and output gain. The limits on the antenna size and efficiency are first discussed by [107].

Hansen and Best [115] have shown that the lower bound of the radiation quality factor *Q*—which measures how many times the current passes through the antenna circuit—depends on the efficiency η:

(8) $$Q\geq \eta \left(\frac{1}{k^{3}a^{3}}+\frac{1}{ka}\right),$$

where *a* is the radius of the spherical radiation and $k=\frac{2\pi }{\lambda }$.

It is pertinent to note the trade-off between antenna size and radiation frequency and antenna designers need to have an optimized design methodology so as to maximize the efficiency while minimizing the size of the antenna at the same time. The *Q*-factor of an antenna can be described as follows:

(9) $$Q=\frac{CF}{BW},$$

where CF is the central frequency and BW the bandwidth. Hence, in order to achieve a higher value of *Q*-factor, the bandwidth has to be narrow and vice versa. Similarly, reducing the size of the antenna decreases *Q* and so the bandwidth tends to increase. It is significant to note that since the product of bandwidth and antenna gain remains constant, increasing the bandwidth would cause a decrease in the gain. Hence, a careful consideration is needed to analyze the trade-off among these antenna design parameters. The fundamental challenge in this case is to design the miniaturized antennas for the least performance degradation. The simplest way to achieve this could be by using high dielectric constant materials exhibiting extremely low losses which enables trimming the antenna size due to the reduction in the size of guided wavelength but also exhibits narrow bandwidth and low gains caused by the excitation of surface waves [116]. However, there could be several other approaches on how the miniaturization could be achieved (such as slits, shorting posts, slots, or geometric optimization, etc.) [117].

#### 6.1.2. Polarization

Antenna polarization is one of the key aspects as regards its design that actually refers to the polarization of the radiated fields of an antenna which could be classified into *linearly polarized* and *circularly polarized* antennas. The later antennas are more prominent in the context of RF energy harvesting due to their capability to radiate and receive RF energy in any plane without high losses [118]. It also helps in improving the power conversion efficiency by reducing the polarization mismatch losses. However, the method employed to accomplish circularly polarized radiations appears to be a limitation as it results in narrow bandwidth [119]. The methods used to accomplish the circular polarization include corner truncation, slits, spur lines, stub-loading, and cross shaped slots in the antenna design. Ref. [120] reports a design incorporating an annular ring around a circular patch for circular polarization that achieves a high conversion efficiency in the order of 25% at 0 dBm input but which starts decreasing drastically at −20 dBm. As a limitation, it employs thin and flexible substrate that tends to suffer from bending losses which ultimately affects efficiency [120].

#### 6.1.3. Harmonic Rejection

Several non-linear components in rectenna design cause the harmonics of fundamental frequencies. These harmonics re-radiate to cause electromagnetic interference with the antenna transmission and the associated rectenna circuit leading to a the degradation of the performance of the overall circuit. Low pass filters need to be added between the antenna and rectification circuit to overcome this issue but at the cost of increase in size and complexity [121]. Harmonic rejection is more important for high-power RF energy harvesting cases as harmonics are more severe in the high-power range. A range of antenna design approaches [122,123,124,125] have been proposed (also known as Filtennas) with the harmonic rejection aiming to maximize the received power on a specific frequency and trying to suppress harmonics with the help of structural modifications (such as stub, slit, and Defected Ground Structures). For example, Razavi et al. [126] present a comprehensive study on the Harmonic Rejection Mixers (HRM) that overviews the HRM operation principles and design issues with the intention to minimize the amount of high frequency filters in the rectenna design and the continuously increasing demand for wide-band radios. Moreover, Forbes et al. [127] design an HRM with programmable frequency which enables multiple effective frequencies in a single HRM without modifying the analogue signal path. A dissertation [128] also throws light on the novel architectures for HRM to achieve a refined level of harmonic rejection by reducing the mismatching sensitivity.

#### 6.1.4. Reconfigurability

Polarization and frequency diversification in wireless systems instigated the development of re-configurable antennas in RF energy harvesting systems [118]. This can be accomplished by modifying the antenna structure which causes a change in its physical properties that helps bandwidth enhancement and size reduction as only a single antenna is able to operate at different frequencies [129]. To this end, Shao et al. [130] attain a wide impedance match employing a two-by-two phase array where beam steering was achieved modifying the feed position in a mechanical way. They control the phase difference between the radiating elements by adjusting the wave path within the transmission line. A recently published survey on re-configurable antennas [131] nicely outlines the switching and implementation techniques with multi-mode and cognitive radio operations. The authors argue that electronic switches are among the popular options to design re-configurable antennas because of their ease of integration, efficiency, and reliability. Another comprehensive survey [132] identifies re-configurable antennas most suited for today’s wireless systems because of their characteristics such as multi-functional capabilities, good isolation, minimized volume requirements, sufficient out-of-band rejection, and low front end processing efforts without a filtering element, to name a few. The authors survey the recently proposed re-configurable antenna designs that are suitable for a broad variety of use cases of wireless communications including, but not limited to, 4G/5G, Ultra Wide Band (UWB) communication, and Multiple-Input Multiple-Output (MIMO) systems. Finally, various proposals have been summarized in Table 6 taking into account a range of antenna design parameters such as polarization technique, harmonic rejection, operating frequency, bandwidth, antenna gains in terms of percentage efficiency, and re-configurability that have been discussed throughout Section 6.1.

### 6.2. Single and Multi-Antenna RF Harvesters

#### 6.2.1. Single Antenna RF Harvesters

As the antenna design involves a range of constraints (such as space and costs an other considerations described above), the research community has striven to optimize the operation of a single antenna design operated at one or several frequency bands as a part of RF power harvester. Omni-directional antennas are one of the obvious choices for RF energy harvesting due to their simple design and 360${}^{\circ }$ coverage to receive and handle the radiating signal in conjunction with a reasonable conversion efficiency. Some of the antennas in this category achieve high efficiency on single frequency (e.g., 2.45 GHz) [138,139], while dual-band [140,141,142,143,144,145], broadband [146,147,148,149,150,151], and multi-band antennas [152,153,154] are also reported in the literature and will be outlined throughout this subsection.

##### Dual-Band Antennas for RF Energy Harvesting

Sun et al. [140] present a dual band antenna for harvesting ambient energy while operating on Global System for Mobile communication (GSM)-1800 and Universal Mobile Telecommunication Systems (UMTS)-2100 frequency bands, that is based on a broadband quasi-Yagi antenna array with higher gains and bandwidths. Moreover, the dual-band rectifier they propose effectively improves the conversion efficiency for ambient RF power. The results demonstrate a conversion efficiency of 40% and an output of 224 mV on a 5 kΩ resistor with an input power density of 455 μW/m${}^{2}$. Similarly, Bakkali et al. [141] propose a dual-band antenna design for ambient RF energy harvesting from commercial broadcasting stations. The authors present several antenna designs aiming to improve the power conversion efficiency of the overall rectenna system as compared to 2.45 GHz and 5 GHz (i.e., WiFi bands). The designed dual band antenna is suitable to be integrated with RF energy harvesting systems and is capable of feeding a range of sensor nodes installed in harsh industrial environments.

The same authors developed a design for a receiving antenna [142] to more effectively harvest the RF energy due to the performance optimization to increase the antenna gains in WiFi bands such as 2.45 and 5 GHz. The return loss in this case goes below −29 dBm for 5 GHz frequency band and the radiation shape exhibits omni-directional patterns. Another work [143] presents a Koch-like dual-band antenna design for RF power harvesting which targets GSM-900 and WiFi frequency bands to harness energy. The simulations and the extensive measurements carried out herein to assess the functionality and performance of the proposed approach justify the superiority of the dual-band Koch-like antenna design over conventional counterparts.

Similarly, Singh et al. [144] propose an open ended microstrip based compact dual-band antenna targeting UMTS and RFID signals. This antenna design has a Sierpinski fractal geometry aiming to cover 2.1 GHz and 5.8 GHz bands. A harmonic suppression method is employed in order to improve the RF-DC power conversion efficiency in both the supportive bands. The results demonstrate that a power conversion efficiency of 79% and 86% are achieved for a 1 kΩ on 2.1 GHz and 5.8 GHz, respectively, which compares well to the state of the art in the area. Finally, Jei et al. [145] propose a circularly polarized dual-band antenna design along with a compact rectifier for RF power harvesting systems. The prototype of a compact rectifier is presented and measured with dual band targeting 0.9 and 2.45 GHz where the conversion efficiency was achieved as 40% at 0.9 GHz and 39% on 2.45 GHz, respectively.

##### Broadband Antenna for RF Energy Harvesting

A range of proposals for broadband antennas have also been presented in the recent past. For example, Arrawatia et al. [146] propose a broadband omni-directional antenna for RF power harvesting offering a bandwidth from 850 MHz to 1.94 GHz which is capable of receiving horizontal and vertically polarized radiating waves with stable pattern for the whole bandwidth. A power conversion efficiency of 60% and 17% are achieved for a 500 Ω load operating at 980 MHz and 1800 MHz, respectively. Consequently, a voltage of 3.76 V for an open circuit and 1.38 V across 4.3 kΩ load is achieved at 25 m distance. Similarly, Song et al. [147] propose a broadband antenna for ambient RF energy harvesting covering the broad spectrum from 1.8 to 2.5 GHz. The authors also design an impedance matching circuit towards improving the performance and efficiency of the overall rectenna for low input power levels. The proposed dual band antenna has a built-in harmonic rejection mechanism to further refine the antenna efficiency. It offers a measured sensitivity of −35 dBm which leads to the conversion efficiency of 55% on the input power of −10 dBm. The results demonstrate that the output power offered by this design is better than the other proposed antennas with similar size and ambient conditions.

Another work [148] presents the design of a broadband antenna for RF energy harvesting supporting a broad spectrum of 900 MHz to 3 GHz. The authors study various issues related to RF design and performed several experiments to improve the efficiency of the rectenna circuit. The results presented herein exhibit that the power of −20 dBm could be harnessed through this kind of system. The connection orientation is also studied by examining both the serial and parallel connection of two RF harvesters compared to a single system. Moreover, Singh et al. [149] propose another compact broadband antenna for RF power harvesting applications which employs a Graphene Field Effect Transistor technique to enhance the impedance bandwidth of the rectifying circuit aiming to cover a frequency range of 22.5–27.5 GHz. The proposed antenna is capable of attaining a conversion efficiency of 80% at 5 dBm for the 5 kΩ load yielding an output voltage of 6.8 V.

Moreover, Shi et al. [150] present a compact broadband antenna for ambient RF harvesting with great bandwidth and matching characteristics. The authors also investigate a rectifier with a single stub matching network for improved impedance matching and RF to DC conversion efficiency even with low input power. A series of simulation and measurements are performed to gauge the performance of the proposed antenna and rectifier where the taken measurements support the simulation results. The results argue that the highest conversion efficiency is recorded as 64% at 0 dBm where the achieved output voltage is 1.5 V. A design of a coplaner waveguide-fed broadband antenna is presented in [151] for RF energy harvesting applications, that was designed to operate at 5.8 GHz ISM band. The antenna prototype was designed using poly-tetrafluoroethylene dielectric material which could achieve a peak antenna gain of 5.85 dBi operating on a 5.8 GHz band. The simulation results argue about achieving the maximum RF to DC power conversion efficiency of 88% at the load of 1 kΩ.

##### Multi-Band Antennas for RF Energy Harvesting

Multi-band antennas have also been studied for many years due to their configuration flexibility, compact size, and leverage to operate on several frequency bands through a single antenna design. For instance, Song et al. [152] developed the concept of a six band dual circularly polarized antenna with an improved impedance matching technique which is useful to maintain the performance level of a rectenna in varying power conditions. The proposed multi-band antenna has a wide bandwidth (i.e., 550 MHz to 2.5 GHz) and an annular ring structure and a feeding technique was taken into account to optimize the size and performance of a multiband antenna. Similarly, the optimal load of 10–75 kΩ is used for a constant power conversion efficiency. The results show that 26 and 8 μW is the maximum harvesting DC power in outdoor and indoor environments, respectively.

Another work [153] proposes a multi-band dual polarized antenna for RF energy harvesting which employs a proximity coupled arrangement to improve the impedance bandwidth so the complete C-band (4–8 GHz) could be covered. The proposed design is compact and dual circularly polarized at three different bands at −10 dB impedance bandwidth. It is evident from the results that a conversion efficiency of 84% could be achieved at 5.76 GHz. Finally, Chandranwanshi et al. [154] present the design of a triple band differential rectenna for RF harvesting which was designed to operate in UMTS frequency spectrum (i.e., 2.1 GHz), WiFi (i.e., 2.4 GHz), and WiMAX (3.3–3.8 GHz). A multi-band slot antenna is employed to design the proposed rectenna with an antenna gain of 7, 5.5, and 9.2 dBi is attainable at 2, 2.5, and 3.5 GHz, respectively. The measurement results demonstrate that a maximum power conversion efficiency of 53%, 31%, and 15.5% could be achieved while operating at 2, 2.5, and 3.5 GHz, respectively.

#### 6.2.2. Multi-Antenna RF Harvesters

The single antenna transmitters covered throughout the last subsection generally suffer from a reduced transfer efficiency as they generate omni-directional radiations of transmitted RF signals. This reduction in transmission efficiency demands for advanced multi-antenna RF harvesting techniques (such as beamforming) to attain spatial multiplexing which yields improving the efficiency of RF power without additional bandwidth or increased transmit power.

The first effort towards multi-antenna RF powered networks for evaluating the throughput performance for energy beamforming was done by Haung et al. [155]. The authors consider a network consisting of a hybrid Access Point (AP) with multiple antennas and a single user where the user has no consistent power supply and the energy is harvested by the broadcasted signals by the AP. They also contribute by deriving closed-form expressions for the delay-limited and delay-tolerant transmissions which are validated through simulations. Finally, the impact of a range of parameters (such as transmit power, harvesting time, throughput, and the number of antennas) is evaluated. Another work [156] proposes the idea of taking into account downlink non-orthogonal systems where the Base Station (BS) is equipped with multiple antennas and caters Relay User (RU) and Far User (FU). Here, the RU harnesses energy from the RF signals received by BS to accomplish relay operation for FU. The authors consider three different types of relaying protocols at RU (such as amplify-forward, decode-forward, and quantize-map-forward) employing beamforming and random antenna selection strategies at BS and RU. The closed form expressions are further derived for the outage probability of relay protocols and antenna strategies.

Huang et al. [157] evaluated the performance of transmit antenna selection schemes for a decode-and-forward RF energy harvesting relay cooperation network. The authors present an idea of power-splitting scheme where the relay harvests some amount of energy from the received signals and then employs the same energy to forward the signal to the destination. The authors also derive the analytical expressions for outage probability (as presented in [156]) and then tractable asymptotic outage probability of the network with different transmit antenna selection schemes in an effort to characterize coding gain and diversity order in higher Signal-to-Noise Ratio (SNR) regimes. The simulation results clearly demonstrate the following three aspects: the second sub-optimal scheme relatively achieves a comparable performance with respect to the optimal scheme with the reduced implementation cost, the relay location is critical to outage performance and optimal power-splitting rate, and the feedback delay is vital towards evaluating the diversity order.

Moreover, Minhong et al. [158] propose the employment of multiple harvesting antennas in an effort to enhance the amount of energy harvested from an energy harvesting device in a given space. The authors used four different cooperating antennas installed in less than twice the square area required by a single antenna and the achieved results identified the suitability of this method for improving the amount of harvested energy. Lastly, Mrnka et al. [159] surveyed different types of antennas for RF power harvesting. The authors identified the following four different types of antennas and marked them suitable for RF energy harvesting: patch antenna, dielectric resonator antenna, modified inverted F antenna, and slot antenna. These antennas were compared on the basis of dimension, reflection coefficient, radiation patterns, and conversion efficiency.

### 6.3. Matching Networks

Impedance matching circuits are an essential component of RF energy harvesting systems and are crucial to the performance optimization of the overall harvesting system. There are a number of techniques for signal modeling to estimate the behavior of devices using linear equations to design impedance matching circuits (e.g., low noise amplifiers). However, these techniques are not suitable to use with RF power harvesting systems because of a very large input signal and the absence of DC bias which leads to the degradation of power conversion efficiency caused by impedance mismatch between rectifier and antenna [160].

Hence, a robust impedance matching network design is desired for achieving a significantly greater power conversion efficiency that has received much attention from the research community. For instance, Hameed et al. [161] propose a systematic technique for developing impedance matching circuits of RF power harvesters towards optimizing the output for a number of input power levels. The experiments demonstrate that the highest amount of harvested energy in case of a fixed input power is achieved when the transistors are biased with stable DC operating voltage. Similarly, the authors also propose a selection strategy for matching networks in the case of variable power levels in order to maximize the expected harvested energy based on the Probability Density Function (PDF) of a given input power level. The proposed RF power harvester demonstrates a maximum power conversion efficiency of 32% at −15 dBm and capable to provide an output voltage of 3.2 V to a load of 1 MΩ.

Similarly, Felini et al. [162] propose an improved dynamic impedance matching network scheme for RF energy harvesting systems. The RF input required for a standardized operation in this scheme is −10 dBm allowing a distance of 1.5 m from a source of 30 dBm. The proposed system is fabricated employing FR4 substrate using discrete components that is able to power general purpose devices by converting RF energy to a DC voltage. The results clearly exhibit the capabilities of the system with optimized impedance matching and received RF power up to +5 dBm. Finally, Agrawal et al. [163] present an analysis of different matching circuit techniques towards realizing efficient RF energy harvesting systems. The authors present RF harvesting systems with three different approaches: resonators, number of multiplier stages, and low pass filters. Resonators are important in improving the amplitude of input signal and are capable of enhancing the performance by up to 30 times. The L type network serves as a resonator as well as matching circuit in the proposed system at resonant frequency and yields maximum power conversion efficiency of 79% on a load of 50 kΩ at an input power of −10 dBm.

### 6.4. Rectifiers/Voltage Multipliers

Rectifiers (also known as voltage multipliers) are another significant subsystem in the RF energy harvesting systems as they are responsible for converting ambient RF harvested power into a useful DC voltage that can be fed to a load. Thus, the performance of a RF energy harvesting system usually depends on both the quality of receiving antenna and the efficiency of a rectifier. It is pertinent to note that the nonlinear component is the core for converting RF signals into DC power. Based on their topological structure and symmetry property, rectifiers could be classified as single-ended (i.e., voltage output) topology [164,165,166] and differential topology (i.e., full wave differential precision) [28,167,168] rectifiers to rectify the higher voltages. The rectifiers used in RF energy harvesting circuits could also be classified based on the frequency bands they cover (such as dual band [169,170,171] and triple band [172,173,174] rectifiers).

There have been a number of proposals for rectenna circuit architectures for RF energy harvesting described throughout this section from single to multi-band rectenna and multi-antenna architectures with different power conversion efficiencies, as shown in Table 7. However, there are several open issues and prospective research directions when it comes to rectenna design. First, Antenna design with maximum power transfer efficiency is still a challenging task with plenty of variables involved. Second, the choice of a best polarization technique for optimum power conversion efficiency is another open issue. Third, antenna orientation significantly influences the performance and, hence, the selection of best orientation of the antennas is inevitable for optimum power conversion efficiency.

## 7. Medium Access Control Protocols for RF Power Harvesting

A Medium Access Control (MAC) protocol defines the way the devices access the medium and, thus, they communicate with each other. A common access way is the carrier-sense multiple access (CSMA) where a device checks if there is an ongoing transmission before starting its own transmission on the same channel. If the channel is occupied, the transmission is postponed—usually for some random time—until the node tries again. Another common way of accessing the medium is the network coordinator to give to each node a usually unique time slot and/or frequency (channel) to perform transmissions. This technique is called Time-division multiple access (TDMA) and Frequency-division multiple access (FDMA) for time slots and frequencies respectively. Combinations between CSMA, TDMA, and FDMA solution may exist.

The choice of an appropriate MAC protocol plays a critical role in the design and performance of RF energy harvesting systems. As communication primitives are known to be responsible for a large portion of energy expenditure in a wireless sensor node quota spent to satisfy the demands of different communication primitives, the design of the MAC protocol is crucial to ensure appropriate sleep intervals for RF energy harvesting and energy optimal operations without impacting on quality of service constraints for communications [175]. Many research works in this space exist investigating the design and evaluation of MAC protocols with energy harvesting support ([176,177,178] to mention some notable ones). This section focuses on reviewing the MAC protocols designed for RF energy harvesting systems. Table 8 summarizes the main features of the protocols described in the next paragraphs.

Kim et al. [179] propose a MAC protocol to adaptively manage the active time of the nodes according to the amount of energy they have harvested. The solution, which is called EA-MAC, is based on the unslotted CSMA/CA algorithm of IEEE 802.15.4. Unslotted CSMA is a commonly used version of CSMA where no time synchronization exists. If a node has enough energy stored in its capacitor (i.e., above a defined threshold), the node wakes up to assess the status of the communication channel and transmit a single packet of data. The random back-off time integral to the protocol design depends on a number of factors. These include the number of channel assessments, a (constant) maximum back-off time, and a fairness co-efficient based on the actual and the average energy harvesting rate of all harvesting nodes in the network. However, the authors do not provide detail as to how the latter is communicated to the nodes. The simulation results show that EA-MAC can achieve high levels of fairness compared to the version without the fairness co-efficient. The authors also present a prototype in an extended work [180].

Naderi et al. [181] present a medium access protocol developed to enable on-demand energy transfer for energy constrained devices. The protocol is called RF-MAC and its operation is divided into three phases during which: (a) A node—whose energy is below a threshold—broadcasts an energy request and one or more transmitters respond. (b) The node decides about how much energy needs to be provided by the transmitters based on its own needs and the information it got from the neighborhood. The transmitters are divided into two groups to maximize the energy efficiency through constructive interference. This is achieved by assigning to the transmitters two slightly separated energy transfer frequencies. In the third phase, the protocol decides to give higher priority to the energy request packets than the data packets for accessing the channel. In this way, nodes with higher residual energy have a higher access priority for data communication. It is shown through simulations and experiments that the proposed protocol achieves higher data throughput compared to the conventional CSMA approach.

Nguyen et al. [182] propose a IEEE 802.15.4-based network MAC layer to harvest energy from ambient LTE signals. The authors assume that the nodes can harvest energy using broadcast transmissions of the downlink LTE channel. Using the residual energy of the nodes, the QoS requirements, and the broadcasted energy, a centralized solution is proposed to adapt the active time of the nodes. The results presented by the authors show high performance gains in terms of energy efficiency. However, a flat RF to DC energy efficiency factor is assumed for all the nodes which is far from reality.

Ha et al. [183] extend the conventional IEEE 802.11e MAC into a harvest-then-transmit-based MAC. It is assumed that the nodes perform transmissions at the same frequency bands with the RF energy emission. Thus, a new medium access method is required to avoid collisions. The authors first present a performance analysis based on a Markov chain model and steady-state probabilities and, at a second stage, determine the maximum achieved energy harvesting rate. The authors’ aim is, despite the new MAC layer, to have backward compatibility with the typical WiFi standard. The simulation results show higher energy harvesting rates when only a limited number of nodes are present. It is seen that the energy rate decreases rapidly with higher number of nodes due to the shorter charging periods available for each node. However, based on the results presented in the paper, the proposed method seems to perform as well as the time-slotted solution of [184] and is seen to outperform the RF-MAC approach [181] and a distributed opportunistic scheduling scheme [185].

Kim et al. [186] propose a new MAC layer to support power emission of dedicated chargers over a contention-based channel access network. The authors’ main objective is to prevent simultaneous data and power emissions over the same channel that could potentially lead to collisions and network disruptions. As a consequence, the design allows energy beacons to be transmitted only when a charger finds the channel idle. The presented simulation results show an improvement from 20% to 150% in terms of harvested energy compared to RF-MAC [181]. However, as with some of the previous works, the results are based on ideal path-loss conditions while the RF-to-DC conversion efficiency is not taken into account.

Unlike the previous CSMA-based approaches, Ju et al. [184] propose a “harvest-then-transmit” approach where a dedicated RF transmitter transfers energy to a set of nodes. The nodes use the uplink transmissions to transmit data packets once they have got enough energy from the transmitter. The uplink transmissions follow a TDMA MAC. The authors deal with the theoretical background of finding optimal joint allocation time for downlinks (RF energy) and uplinks (data). The analysis reveals a fairness issue for the distant nodes because they have to transmit with higher power but, at the same time, they obtain less energy from the transmitter. To address this issue, the authors revise their algorithm to find throughput-fairness trade-offs.

## 8. Limitations, Open Issues, and Future Research Directions

The literature covered in the previous sections focuses on a number of tenets related to the performance of RF energy harvesting systems and describes recent advancements in these areas. However, there are numerous technological limitations and open issues that need to be tackled or mitigated and are described in the following paragraphs. Future research directions for many of the topics covered throughout this manuscript are summarized in Table 9. In addition to the area-specific challenges presented herein, this section highlights additional challenges of a more general nature which are crucial to the performance of RF powered systems.

### 8.1. Limitations

The reduction in available energy density in line with the propagation distance is one of the major limitations to be considered when developing RF power harvesting systems. The inverse square law (i.e., $I\propto (1/d^{2})$) states that the power density of the RF signals declines proportionally to the inverse of the square of the propagation distance between transmitter and receiver and 1 W is the maximum transmitter power output in EU region in compliance with the Federal Communications Commission (FCC) local regulations. In addition, FCC has capped the maximum Effective Isotropic Radiated Power (EIRP) emission up to 4 W equivalent isotropic radiations, whereas [187] indicates that only a distance of 15 m could be supported to achieve an energy transfer rate of 5.5 μW considering a 4 W power source.

Miniaturizing the sensor design is also of critical significance in the presence of RF harvesting circuits as it should be comparable to the size of a battery powered sensor device. As described in Section 6, the antenna, matching network, and rectifier are the integral components of an RF power harvester and the antenna size has a decisive role in determining the harvesting density so it becomes even more pertinent to reduce the size of the devices embedded to the designed sensors while improving harvesting efficiency. Thus, decreasing the circuit (sensor) size and simultaneously improving the harvesting efficiency of the circuit are two different goals and it becomes challenging to achieve both at the same time.

The sensitivity of an RF harvesting circuit (i.e., the minimum power required by the harvester to start the harvesting process) also plays a key role in performance optimization by improving conversion efficiency. There can be a significant difference in the sensitivity of the data receiver component when compared to that of RF power harvester. As a result, if the receiver is located far away from the transmitter, it may be able to decode the information but can not effectively extract the energy from an RF signal from the same transmitter. Consequently, Simultaneous Wireless Information and Power Transfer (SWIPT) techniques do not perform well and efforts are needed on improving the sensitivity of RF harvesting circuit so as to enhance the conversion efficiency at different distances.

### 8.2. Open Issues and Mitigation Actions

Impedance mismatch, which can be defined as the phenomenon caused due to the difference of the input resistance and reactance between the rectifier and the antenna, is also an important concern in RF power harvesters. Consequently, the receiver antenna is unable to transfer all of its harvested power to the rectifier that is one of the most common causes of degradation in power conversion efficiency. Hence, the research community should focus on developing some efficient and smart circuit design techniques to effectively minimize/mitigate this impedance variation and develop system level design activities for antenna and rectenna, incorporating not just the design of the antenna but also considering the impedance characteristics of the antenna.

Energy loss in a non-line of sight environment is another issue that deserves attention. A substantial amount of energy loss is anticipated coming from a source to a harvester in this case. So, if the transmitter (i.e., the energy source) is to provide energy to multiple receivers at the same time, line of sight at appropriate distance should be maintained. This becomes particularly relevant for sensors operating in a mobile environment where the mobility may severely impact the harvestable power densities when transmitters and/or harvesters are mobile. Reconfigurable intelligent surfaces could help in such an environment through controlling the propagation of electromagnetic signals by changing the electric and magnetic properties of the surface aiming to minimize such losses [188].

A scheduling policy is an important aspect to consider in RF power harvesting systems as it can turn potential EM interference into useful energy by introducing an efficient scheduling policy. Spectrum scheduling could be useful to avoid or mitigate the interference by employing some protocol management techniques such as interference cancellation and alignment of interfering signals. Here, simultaneously mitigating the interference and transferring the energy from transmitter to receiver might be two contradictory goals to be achieved in RF power harvesting that could indeed be challenging as the interference mitigation techniques could impact the energy harvesting magnitudes. Such power management techniques along with an appropriate scheduling policy can result in a significant improvement in energy efficiency.

Finally, an evident issue in the domain of RF-harvesting is that of energy fairness among the nodes. This means that nodes that are distant from the power transmitters receive not only less energy but they also have less chances to compete for the data channel. Even though some of the studies try to resolve this issue by adapting the duty cycle time of the nodes, the problem still remains. The issue could potentially be tackled or mitigated by appropriately placing the transmitters in the spatial domain [189,190,191]. Another issue with most of the proposed MAC protocols is the lack of experimentation and reliance on simulations to characterize energy harvesting networks. Typically results are based on mathematical analysis, computer simulations and modeling—usually assuming free-space path loss—while no real energy conversion efficiency is evaluated in testbeds and physical deployments.

### 8.3. Other Research Directions

There are a number of challenges that led future directions and opportunities in this domain.

The potential amount of harvested energy available from RF power harvesting makes the EM spectrum potentially a valuable resource and opens up opportunities in establishing a competitive market to economically manage and exploit this resource. For instance, wireless charging service providers could be seen as small RF power suppliers contributing towards (partially) meeting the energy demands of nodes over the network. As such, these service providers can decide on pricing of the commodity they provide while guaranteeing the quality of service for the sensor networks of their customers.

Mobility is another key concern in RF power harvesting and information transfer where network sources, sensor nodes, and gateways may all be mobile in nature. This issue becomes even more critical in the case of the time varying nature of RF harvesting and information transmission as the amount of energy harvested is not always the same in magnitude and power density. Appropriate resource allocation can be tricky to be implement in these kinds of systems where the research opportunities exist in achieving dynamic and adaptive resource allocation.

Network coding schemes are another potential topic of research as such schemes can heavily influence the energy efficiency of a wireless system as it enables wireless transmitters to transmit data, information, and energy simultaneously over common frequency bands. This characteristic enables an improvement in RF energy harvesting densities, particularly in case of large-scale networked systems. In such systems, data transmitter and relay nodes can effectively use available time slots for power harvesting when they are idle from a data transmission perspective. The authors [192] suggest that network lifetime gain has the potential to be improved by up to 70% when employing RF power harvesting technology with appropriate coding schemes. A diverse range of network model and coding schemes need to be explored in order to improve the network lifetime. It is still an open issue to investigate whether the RF power harvesting improves the upper bound of energy gains and to what extent.

Finally, it has widely been acknowledged that RF exposure causes heating some materials with finite conductivity [193]. Several studies [194,195,196,197,198] have been conducted on the impact on human health of electromagnetic radiations from mobile devices and cellular infrastructures. The majority of these studies identify EM radio waves as non-harmful for human health. A minority of them [197,198] report some effects on genes when exceeding the upper bound of internationally recognized standards on secure power levels. The impact of RF chargers used for the energy harvesting technologies discussed in this publication on health as these may emit radio waves at high power densities so there is a need to gauge the safety standards while deploying these RF chargers in conjunction with FCC standards.

## 9. Conclusions

With the advent of IoT, exploring different methods of harnessing energy from the environment (such as electromagnetic, photoelectric, thermoelectric, and piezoelectric to name a few) in which sensor nodes are deployed to feed the end devices, has globally become a topic of research interest. All of them have their own sets of pros and cons that determine their suitability and limitations for a unique use case based on their different characteristics and design considerations. RF energy harvesting is one promising solution to achieve network longevity where a sufficient amount of energy could be harvested from both ambient and dedicated RF sources. However, the technological advancements in this area (specifically, harvesting techniques, energy propagation models, rectenna architectures, and MAC protocol design) are progressing slowly and only a limited number of real prototypes have been reported and a number of interesting research challenges are identified. The paper comprehensively surveys the current state of the art in the domain of energy harvesting with a particular focus on RF energy harvesting. The factors that influence the performance of RF energy harvesting systems and a range of harvesting applications (e.g., industrial automation, healthcare informatics, structural health monitoring among many others) are described. The review covers the disparate research activities required for RF energy harvesting including dedicated vs. ambient harvesting sources, evaluation metrics, energy propagation models, rectenna architectures, and MAC protocols for RF power harvesting. There exist a number of open research challenges and future research directions for the new entrants working into RF energy harvesting domain.

## Figures and Tables

**Table 1:** Renewable energy sources along with their power densities and efficiency.

Harvesting Method	Power Density	Efficiency
Solar energy—outdoors	15 mW/cm${}^{3}$-bright sunny day	10–25%
	0.15 mW/cm${}^{3}$-cloudy day	
Solar energy—indoors	100 µW/cm${}^{3}$	
Vibrations (piezoelectric—shoe inserts)	330 µW/cm${}^{3}$-105 Hz	25–50%
Vibrations (electrostatic conversion)	184 µW/cm${}^{3}$-10 Hz	
Vibrations (electromagnetic conversion)	0.21 mW/cm${}^{3}$-12 Hz	
Thermoelectric (5–20 °C gradient)	40 µW-10 mW/cm${}^{3}$	0.1–3%
Magnetic field energy	130 µW/cm${}^{3}$-200 µT, 60 Hz	30–74.4%
Wind energy	65.2 µW/cm${}^{3}$-5 m/s	20–40%
RF energy	0.08 nW-1 µW/cm${}^{2}$	30–88%

**Table 2:** Experimental evaluations for RF power harvesting.

Transmitter	Transmit Power	Frequency	Distance	Harvesting Power (at a Reference Distance)
Isotropic RF transmitter [27]	4 W	902–928 MHz	15 m	5.5 μW
Isotropic RF transmitter [28]	1.78 W	868 MHz	25 m	2.3 μW
Isotropic RF transmitter [15]	1.78 W	868 MHz	27 m	2 μW
TX91501 powercaster transmitter [29]	3 W	915 MHz	5 m	189 μW
TX91501 powercaster transmitter [29]	3 W	915 MHz	11 m	1 μW
King TV tower [30]	960 kW	674–680 MHz	4.1 km	60 μW

**Table 3:** A summary of the state of the art surveys on RF power harvesting.

Reference	Focus	Objectives	Year
Verma et al. [19]	Hardware design issues	– Surveys the literature on the theoretical studies of RF harvesting. – Outlines the possible energy harvesting and energy transfer technologies.	2016
Mukminin et al. [20]	RF energy sources	– Studies the literature on the use of RF energy harvesting. – Outlines a range of antennas suitable for RF energy harvesting.	2020
Lu et al. [21]	RF powered wireless networks	– Overviews the RF energy harvesting network architecture. – Presents circuit design background. – Explores design issues in the development of RF harvesting networks.	2015
Nintanavongsan et al. [22]	Circuit and protocol design	– Studies the fundamental design of RF energy harvesting circuits and protocols. – Discusses the impact of energy replenishment capabilities on circuits and protocols.	2014
Sidhu et al. [23]	energy harvesting sources	– Surveys a range of ambient RF enegry harvesting sources. – Reviews the progress of ambient sources that is useful in designing the RF energy harvesting model.	2019
Soyata et al. [24]	Trade-offs and methodologies on RF harvesting for embedded systems	– Overviews the passive RF energy reception and harvesting circuits. – Discusses RF energy harvesting in the context of embedded systems. – Analysis different combinations of system components.	2016
Tran et al. [25]	Design methodologies and applications for RF harvesting	– Summarizes RF power harvesting technologies to design the system. – Provides discussion on different design alternatives and their trade-offs for RF power harvesting.	2017
Srininvasu et al. [26]	Conceptualization of RF energy Harvesting	– Studies the optimal rectenna architecture for maximizing power conversion efficiency. – Discusses system architecture for RF harvesting networks and applications.	2019
Clerckx et al. [31]	Future Networks With Wireless Power Transfer and Energy Harvesting	– Presents the recent theory, designs, prototypes, and experiments in the area [32,33,34]. – Shows how metamaterials and metasurfaces such as intelligent reflecting surfaces can significantly improve the power transfer efficiency and operational distance [35,36]. – Use of backscatter communications for RF power harvesting systems [37,38]. – Resource allocation and safety constraints for Simultaneous Wireless Information and Power Transfer [39,40]. – A review on fundamental principles of primary PHY attacks, covering jamming, eavesdropping, and detection of covert [41].	2022
This Survey	Application domains and performance determinants of RF powered systems	– Overviews a range of applications of RF harvesting along with their performance requirements. – Studies the factors affecting the performance of RF harvesting systems. – Highlights challenges and future research directions critical to system design and performance optimization.	2022

**Table 4:** Performance requirements for different application domains.

Application Domain	Coverage	Transmission Frequency	Operational Cost	Energy Efficiency	Latency	Network Type
Internet of Things	Varies *	Medium	Medium	Varies *	Medium	WAN
Industrial Automation	Medium	High	High	Low	Medium	WAN
Healthcare Informatics	Low	Low	Low	High	Low	PAN
Radio Frequency Identification	Low	Low	Low	High	Low	PAN
Smart Buildings/Structural Health Monitoring	Medium	Low	Low	High	Low	WAN

* Varies from low to high depending on the type of IoT use cases. PAN stands for Personal Area Networks, WAN stands for Wide Area Networks.

**Table 5:** Power densities of ambient RF power harvesting sources in different frequency bands.

Band	Frequency Range (MHz)	Average Power Density (nW/cm${}^{2}$)	Maximum Power Density (nW/cm${}^{2}$)
DTV [95]	470–610	0.89	460
GSM900 (MTx) [95]	880–915	0.45	39
GSM900 (BTx) [95]	925–960	36	1930
GSM1800 (MTx) [95]	1710–1785	0.5	20
GSM1800 (BTx) [95]	1805–1880	84	6390
3G (MTx) [103]	1920–1980	0.46	66
3G (BTx) [103]	2110–2170	12	240
Wi-Fi [103]	2400–2500	0.18	6

**Table 6:** A chronologically ordered summary of indicative harmonic rejection and re-configurable antennas for RF power harvesters.

Antenna Proposal	Feature	PL *	HR **	Frequency (GHz)	Bandwidth (MHz)	Gain (dBi)/ Effic (%)	RC †
Polarization reconfigurable monopole antenna [133]	Circular patch with reconfigurable feed antenna	1 LP, 2 CP	No	5.07–5.86	0.79	3	Yes
T-shaped slot wideband antenna [134]	T-shaped conductor line connected to path	CP	Yes	2–3.5	1.5	5.5	No
Metamaterial based multiband antenna [135]	Antenna loaded with interdigital capacitor slots	LP	No	OFF: 7–8.5 ON: 3.8–4.2, 5.5–6, 6.8–8.5	OFF: 1.5 ON: 0.4, 0.5, 1.7	OFF: 1.93 ON: 1.78, 1.63, 2.32	Yes
Switchable Filtenna [136]	3-loop resonators in UWB antenna	LP	Yes	OFF: 3.2–11 ON: 3–3.5, 4–5.7, 6.2–11	7	OFF: 4.33, ON: 3.8 96%	Yes
Off center fed dipole antenna [137]	Dipoles are modified into bow tie stubs	CP	No	1.8, 2.5	0.7	3.5	No

* Polarization: CP = Circularly Polarized; LP = Linearly Polarized, ** Harmonic Rejection, † Reconfigurable.

**Table 7:** Summary of power conversion efficiencies achieved on different frequency bands and loads.

Reference	Antenna Type *	Frequency Band	Power Conversion Efficiency, PCE (%)
Mhatre et al. [71]	SB	2.4 GHz	30%
Sun et al. [140]	SB	GSM-1800 MHz and UMTS-2100 MHz	40% @ 5 kΩ
Singh et al. [144]	DB	2.8 GHz and 5.8 GHz	79% and 86% @ 1 kΩ
Arrawatia et al. [146]	BB	850 MHz to 1.94 GHz	60% and 17% @ 500 Ω
Song et al. [147]	BB	1.8 to 2.5 GHz	55% @ −10 dBm
Singh et al. [149]	BB	22.5 GHz to 27.5 GHz	80% @ 5 dBm & 5 kΩ
saranya et al. [151]	BB	5.8 GHz to 5.85 GHz	88% @ 1 kΩ
Singh et al. [153]	MB	C-band (4–8 GHz)	84%
Chandra et al. [154]	TB	2 GHz, 2.5 GHz, and 3.5 GHz	53%, 31%, 15.5%
Hameed et al. [161]	SB	902–928 MHz	31% @ 1 MΩ
Agrawal et al. [163]	SB	900 MHz	79% @ 50 kΩ

* SB—Single Band; DB—Dual-Band; TB—Tri-band; BB—Broadband; MB—Multi-band.

**Table 8:** Characteristics and features of the proposed RF power harvesting enabled MAC protocols.

Ref.	RF Energy Emission	Ambient/Dedicated Harvesting	Medium-Access Method (Radio)	Maximization Parameters	Experimentally Validated
[179]	Constantly	Ambient	CSMA (IEEE802.15.4)	Throughput, Fairness	Yes
[181]	On demand	Dedicated	CSMA	Throughput	Yes
[182]	Constantly	Ambient (LTE)	CSMA (IEEE802.15.4)	Throughput	No
[183]	Harvest-then-transmit	Dedicated (WiFi)	CSMA (IEEE802.11)	Throughput	No
[186]	When no data	Dedicated	CSMA	Harvesting energy	No
[184]	Harvest-then-transmit	Dedicated	TDMA	Throughput, Fairness	No

**Table 9:** Challenges and future research directions in RF power harvesting systems.

Section	Challenges and Open Issues	Future Directions
Evaluation metrics for RF power harvesting	– Choice of optimal operational frequency. – Achieving optimal power conversion efficiency.	– Improvement in the sensitivity of the rectenna system. – Optimizing the DC output power.
Applications of RF energy harvesting	– Energy optimal IoT operation. – Green and eco-sustainable IoT. – Optimal use of small power magnitudes. – Reducing the operational costs of devices.	– Designing smart wearables for healthcare informatics. – Designing battery free sensors for structural health monitoring. – Exploiting RF power harvesting for RFID tags.
Dedicated vs. Ambient RF energy Harvesting	– Small power densities of ambient RF sources. – Higher distances between RF transmitters and harvesters. – Higher CAPEX in case of dedicated RF power sources. – Site selection for installing dedicated chargers. – Antenna & Rectifier design for static ambient RF harvesting	– Investigating the hybrid (ambient & dedicated) RF power sources. – Improving the conversion efficiency of hybrid RF harvesting systems. – Exploiting RF power harvesting for RFID tags. – exploring ways to directly feed ambient RF energy to sensors.
Energy propagation models	– To measure the characteristic signal parameters. – The accurate prediction of the RF signal behavior in real time. – Most of theoretical propagating models are not validated through experiments.	– The analysis of RF signal propagation employing different models. – Estimating the distribution of RF power considering fading effects and path loss. – Improving the precision of the RSSI of an RF signal at harvester side.
Rectenna Architecture for RF energy Harvesters	– Antenna design with maximum power transfer efficiency. – Choice of a best polarization technique for optimum power conversion efficiency. – Selection of best orientation of the antennas for optimum conversion efficiency.	– Proposing new filtennas offering low pass filters for rejecting the harmonic rejection. – Balancing the trade-off between single & multi-antenna design in RF harvesters. – Exploring multi-antenna RF harvesting techniques(e.g., beamforming to attain spatial multiplexing) for higher efficiency.
MAC Protocols for RF power Harvesting	– Energy fairness among the nodes. – Lack of experimentally validated models.	– Design of MAC protocol to ensure the fair energy quota across all the nodes. – RF transmitter localization across the network.
